# Biomimetic Dermal Substitutes for Skin Reconstruction: Advances in Scaffold Design, Biological Integration, and Clinical Performance

**DOI:** 10.3390/jfb17080397

**Published:** 2026-08-12

**Authors:** Bogdan Mircea Măciuceanu Zărnescu, Iulia Ioana Lungu, Adelina-Gabriela Niculescu, Alexandru Scafa Udriște, Constantin Cosmin Baciu, Daniela Anghel, Adrian Radu Rădulescu, Alexandru Mihai Grumezescu, Sebastian Vâlcea

**Affiliations:** 1 University of Medicine and Pharmacy “Carol Davila”, 8, Eroii Sanitari, 050474 Bucharest, Romania; bmaciuceanu@gmail.com (B.M.M.Z.); alexandru.scafa@umfcd.ro (A.S.U.); constantin.baciu@umfcd.ro (C.C.B.); radu_radulescu@umfcd.ro (A.R.R.); sebastian.valcea@gmail.com (S.V.); 2 Lasers Department, National Institute for Lasers, Plasma and Radiation Physics, 409 Atomistilor St., 077125 Magurele, Romania; iulia.lunguu@gmail.com; 3 Research Institute of the University of Bucharest—ICUB, University of Bucharest, 050657 Bucharest, Romania; agrumezescu@upb.ro; 4 Department of Internal Medicine 2, Central Military Emergency University Hospital Dr. Carol Davila, 010825 Bucharest, Romania; daniela.anghel@prof.utm.ro; 5 Department of Medico-Surgical and Prophylactic Disciplines, ‘Titu Maiorescu’ University, 031593 Bucharest, Romania; 6 Faculty of Chemical Engineering and Biotechnologies, National University of Science and Technology Politehnica Bucharest, Gh. Polizu St. 1–7, 060042 Bucharest, Romania

**Keywords:** polymeric biomaterials, skin tissue engineering, dermal scaffolds, wound healing, dermal regeneration, extracellular matrix, collagen-based scaffolds, biodegradable polymers, angiogenesis, 3D bioprinting

## Abstract

Reconstructive surgery still faces various challenges when dealing with the reconstruction of complex skin defects, regardless of their origin (e.g., trauma, burns). Split-thickness autologous skin grafts remain the gold standard; however, this technique comes with several limitations, including donor site morbidity, infection, and scarring. Since extensive research on conventional techniques has not provided sufficient long-term results, there has been a shift toward dermal substitutes made from regenerative biomaterials that can aid wound management and support tissue restoration. This structured review will introduce the challenges posed by complex skin defects, the limitations of autologous skin grafts and conventional reconstruction techniques, and the emerging field of dermal substitutes and biomimetic scaffolds. Moreover, the native dermis is presented as a blueprint for biomimetic scaffold design, along with key principles to consider when engineering a dermal scaffold, from architecture and porosity to angiogenic potential. A critical examination of the main classes of available dermal substitutes and their clinical performance is conducted, highlighting their limitations and the translational gaps in current evidence. Although the current pool of biomaterials still faces challenges in accurately mimicking the properties and architecture of natural skin, future perspectives offer promising alternatives that could overcome these limitations.

## 1. Introduction

### 1.1. Paradigms in Reconstruction Surgery

As a specialized surgical subspecialty, plastic surgery primarily focuses on reconstructive and reparative procedures of the human body. Plastic surgery is a practice that started in antiquity, became in demand in World War I, and has since evolved and advanced technologically with advances in medicine. Recently, improvements in technology have forced substantial shifts in the conceptual frameworks of the medical field. Specifically in reconstructive and plastic surgery, a variety of reconstructive options have become available as a result of field innovation [1].

**Figure 1:**
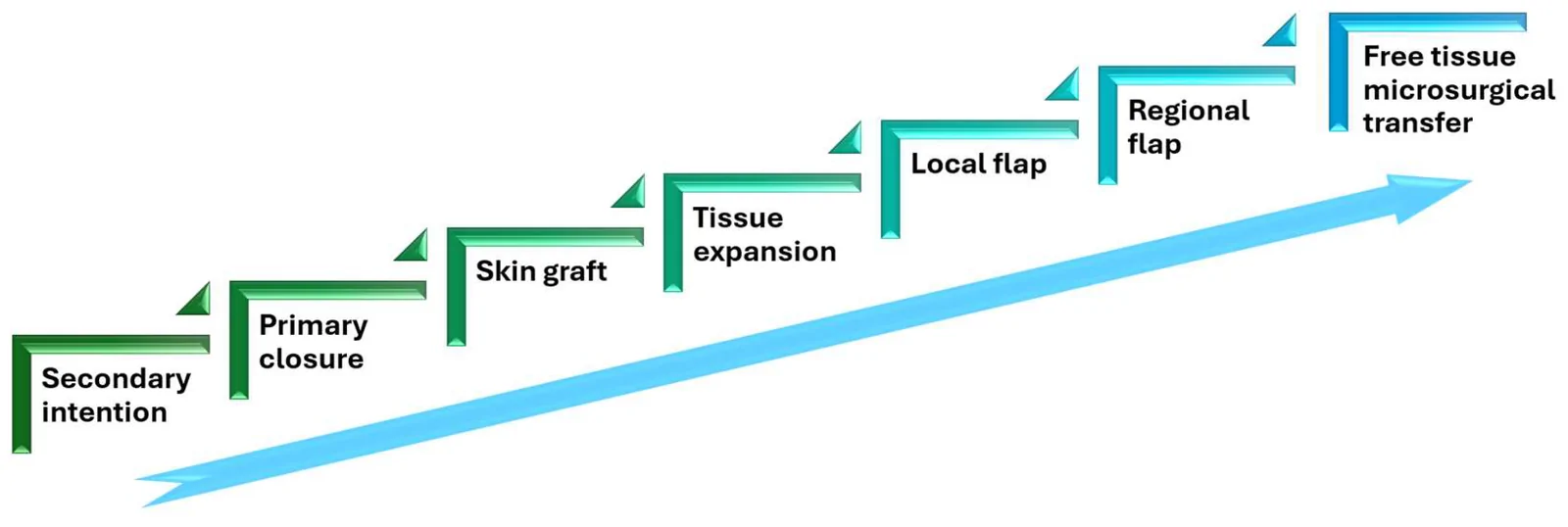
The reconstructive ladder. Created based on information from [1].

When discussing reconstructive surgery, several paradigms have been proposed over the years to address the surgical requirements of individual cases. One of the main paradigms in the field of reconstructive surgery is the ‘reconstructive ladder’—this allows the surgeon to choose the desired procedure, ranging from minimally invasive to complex options [1,2]. The priorities when using the ‘reconstructive ladder’ (depicted in Figure 1) are based on the complexity of the defect/deformation, to attain the highest level of patient safety, which is the guiding principle for plastic surgeons [3,4].

Based on this approach, the priority should be choosing the simplest technique that can cover the defect. However, its disadvantage is that form and function restoration fall second to defect coverage. To be taken into account, a complex reconstruction should be considered; therefore, the next paradigm is introduced. The ‘reconstructive elevator’ was first introduced in 1994 by Gottlieb and Krieger. They emphasize the flaws of the previous paradigm and state that the technique should be selected based primarily on ensuring functional outcomes—therefore, sometimes, choosing a complex reconstructive procedure from the start [5]. Three years later, Mathes and Nahai propose a third, more personalized to patient needs, paradigm, the ‘reconstructive triangle’, which takes into account patient safety, form and function restoration, as well as donor site morbidity minimization [6]. Through the previous paradigms, special consideration should also be given to the surgeon’s experience and skill set; therefore, Wong and Niranjan proposed the ‘reconstructive stages’, which add the development of surgical abilities to the previously known standards [7]. With the evolution of technology and medicine, as well as taking into consideration the socio-economic aspect, the concept of ‘reconstructive matrix’ took root and was introduced in 2010 by Erba et al. [8]. This approach was based on a 3D cubic model that considered technological refinement, surgical complexity, and surgical risk to tailor potential reconstructive approaches. During the same time, Knobloch and Karsen proposed an integral procedure that accounts for innovative techniques such as composite tissue allotransplantation, robotics, regeneration, and tissue engineering, which are part of the reconstructive sequence but rather simultaneous than consecutive—the proposed paradigm was termed ‘reconstructive clockwork’ [9]. As expected, new advancements introduced new solutions for existing problems, and these paradigms kept evolving and improving over the years. The latest paradigm introduced into the literature was by De Francesco et al., named ‘translational reconstructive ladder’. This concept basically merged the classical concepts provided by previous paradigms, while also integrating technological advancements and regenerative medicine [10].

### 1.2. Challenges in Complex Skin Defects

There is no clear definition that can characterize complex skin defects through which they can be classified. Generally, the term ‘complex wound’ is used to describe chronic or acute wounds that pose challenges to the medical team in terms of healing by conventional strategies. However, several factors may contribute to complex wounds: considerable loss of integument, infection, necrosis, or impairment of circulation, and association with systemic pathologies that can affect the normal healing process (e.g., diabetes) [11,12].

There are, however, a multitude of factors that can influence wound healing. These factors can be local, such as lesion depth, infection, peripheral vascular disease, exposure to radiotherapy, constant pressure, and excessive moisture, as well as systemic factors, including metabolic disorders (e.g., diabetes), immunodeficiency, and nutritional deficiencies [13,14] (Table 1).

From a biomedical point of view, the main issue in dermal reconstructive surgery is not necessarily the missing part (for which substitute strategies will be further discussed), but the potential hostile environment that hinders natural regeneration and limits the success of reconstructive strategies (such as grafts, substitutes, etc.).

### 1.3. Limitations of Autologous Skin Grafts and Conventional Reconstruction Techniques

The natural wound healing process consists of four interconnected, overlapping, well-organized phases: hemostasis, inflammation, proliferation, and tissue remodeling. For ideal results, these four phases must follow a specific protocol regarding sequence, timing, and duration. Wound healing impairment occurs when one or more of these phases is interfered with [20].

Complex skin defects or non-healing wounds represent a challenge for modern medicine, and there are several reasons why conventional reconstruction techniques are limited [21]. Managing large dermal defects that occur from burns, chronic wounds, traumatic injuries, or other reasons poses challenges for reconstructive surgery. Dressings, negative pressure therapy, and transplantation are conventional treatments for chronic wounds; however, they come with several drawbacks [22]. For instance, allogeneic or xenogeneic transplantation can induce strong immune responses that can result in rejection. They are rather used as temporary dressings, creating and maintaining a proper environment for the survival and development of autologous grafts [23]. On the other hand, autologous skin grafts are known for their fast integration and low rejection rate. However, the limitations of these grafts include donor-site morbidity and the limited availability of tissue for large cutaneous defects. Due to these limitations, alternative methods for the management and treatment of complex skin defects need to be explored [22].

### 1.4. Emergence of Dermal Substitutes as Regenerative Biomaterials/Biomimetic Scaffolds

The gold standard for wound healing and repair continues to be the use of autologous grafts. Nevertheless, when the area is simply too large for autologous grafts to be considered, alternative techniques for dermal reconstruction need to be regarded. Tissue engineering and the advancement of dermal substitutes could potentially and efficiently overcome these limitations [24,25]. Bioengineered skin substitutes are typically made from either synthetic or biologically derived materials (biocompatible scaffolds) that can mimic both the structure and function of natural skin and serve to promote wound healing, provide temporary coverage, assist permanent skin replacement, and reestablish barrier function [26]. To aid tissue integration and regeneration, these substitutes can use patient-derived cells or growth factors [27]. These biomimetic scaffolds are mechanically stable and flexible, promoting an ideal environment for cell growth and encouraging cellular adhesion, proliferation, and differentiation, thereby aiding tissue regeneration [28]. Even though these substitutes are still undergoing optimization and refinement, they serve as valuable alternatives to classical grafting methods for severe skin loss cases.

The scope of this work is to provide a comprehensive review of biomimetic dermal substitutes for skin reconstruction, starting with the structure of the native dermis and progressing to different classes of biomimetic scaffolds, their clinical use, and performance. The mechanisms by which dermal substitutes are integrated and promote tissue regeneration will be discussed, as will the various clinical applications for which they can be used. Finally, the current limitations the field still has to overcome will be summarized with potential future directions in biomimetic dermal engineering.

### 1.5. Search Methodology

This structured literature review was accomplished by analyzing English-language publications from PubMed, Google Scholar, and Web of Science databases. The search strategy employed the following terms (and combinations of): ‘reconstructive surgery’, ‘skin defects’, ‘conventional reconstruction techniques’, ‘dermal substitute’, ‘skin substitute’, ‘biomimetic scaffolds’, ‘tissue engineering’, ‘burn reconstruction’, ‘traumatic soft tissue defects’, ‘oncologic reconstruction’, ‘chronic wounds’, ‘complex skin defects’, ‘prevascularization’, ‘3D bioprinting’. The research was not strictly limited by publication date; however, most selected articles were published in the last 10 years. Priority was given to systematic reviews, randomized clinical trials, clinical trials, and meta-analyses.

## 2. Native Dermis as a Blueprint for Biomimetic Scaffold Design

Before going into depth regarding biomimetic scaffolds and the use of tissue engineering for regenerative processes, a fundamental understanding of the underlying structure and function of the natural tissue is required. Simply put, the skin is the body’s largest, most complex organ, serving as a barrier between the external environment and the body, protecting it from environmental stressors. Another important role is preventing water loss and regulating body temperature. Structurally, it is composed of two tissues—the dermis and the epidermis, which have distinct anatomies and functionality. Other skin structures, also called appendages, include hair follicles and sebaceous and sweat glands, which are of ectodermal origin and are critical for the proper functioning of the skin [29]. The deepest layer of the skin is called the hypodermis (subcutaneous adipose tissue) and is mainly composed of adipocytes and loose connective tissue (collagen and elastin); it loosely anchors the skin to the underlying muscle and bones. It plays a key role in energy storage, shock absorption, and thermal insulation [30].

**Figure 2:**
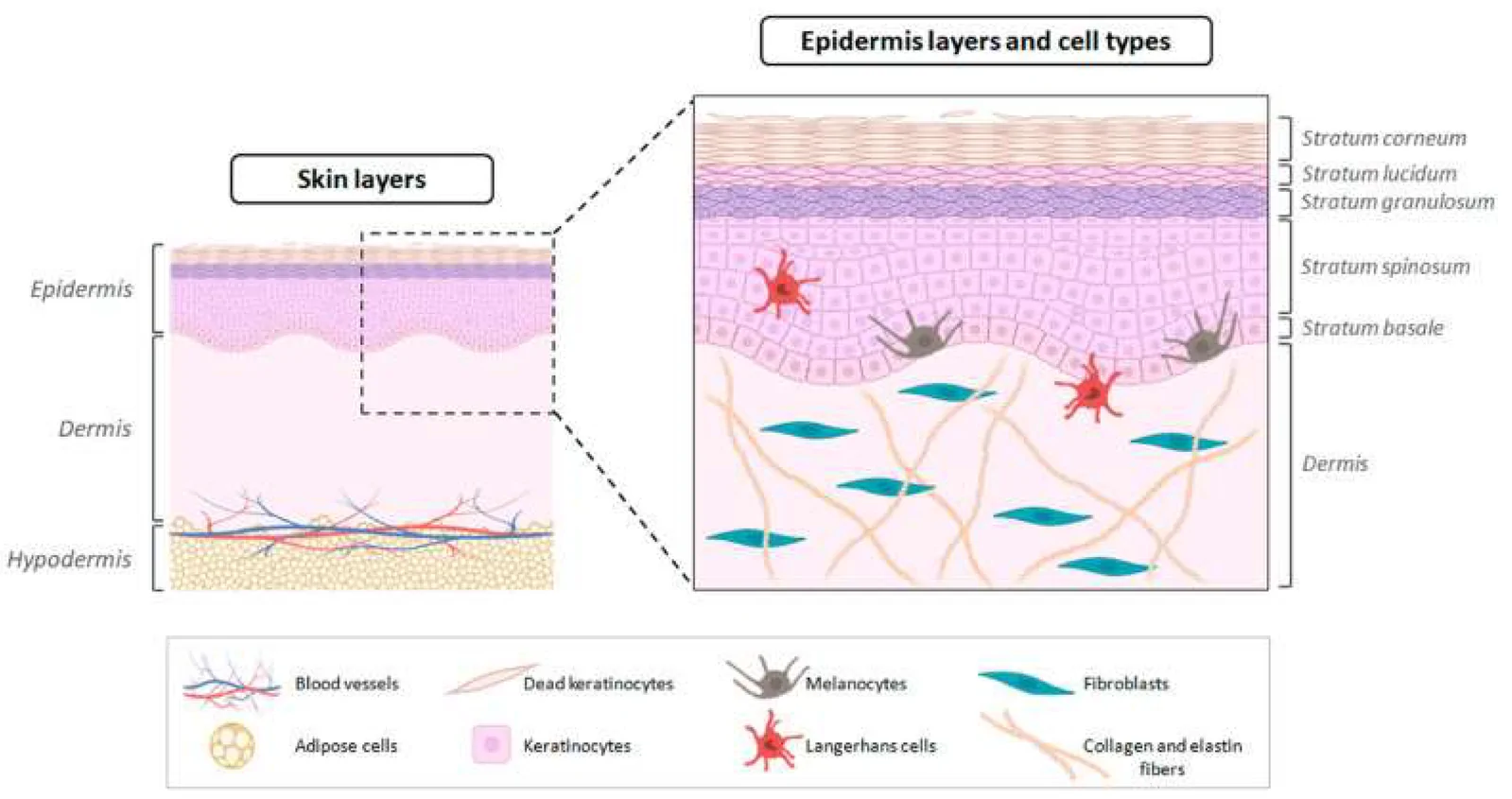
Schematic representation of skin layers, with focus on epidermis and main cell types. Reprinted from Ref. [32].

The epidermis is the outermost layer of the skin and is largely composed of a specific type of epithelial cells, namely keratinocytes. Pigmentation, sensory, and immunological functions are carried out by resident cells, including melanocytes, Merkel cells, and Langerhans cells, respectively. Structurally, the epidermis is divided into distinct strata (layers), shown in Figure 2. Apart from the represented strata, there is a fifth layer—stratum lucidum (transparent/clear layer) found between the granulosum and corneum strata, generally composed of a couple of layers of cells, the main representatives being keratinocytes [31].

The renewal of the epidermis takes place in the deepest layers of the basal layer, where progenitor cells (undifferentiated keratinocytes) are found; the renewal process occurs continuously. The keratinocytes formed in the basal stratum move upward toward the surface, where they undergo desquamation. During this ‘travel to the surface’, the keratinocytes undergo a process of differentiation through which their structure and physiological activity change. The final step is called terminal differentiation and results in corneocytes (anucleate keratinocytes), which comprise the stratum corneum—the main constituent of the protective skin barrier. Since it is an avascular tissue, nutrition and oxygenation of the epidermis are contingent on the dermis [29,31].

Separating the epidermis from the dermis is a thin layer (<200 nm across) named the cutaneous basement membrane. Despite its size, the cutaneous basement membrane is responsible for the adhesion between the epidermis and the dermis, achieved by its resident proteins and glycoproteins [33].

**Figure 3:**
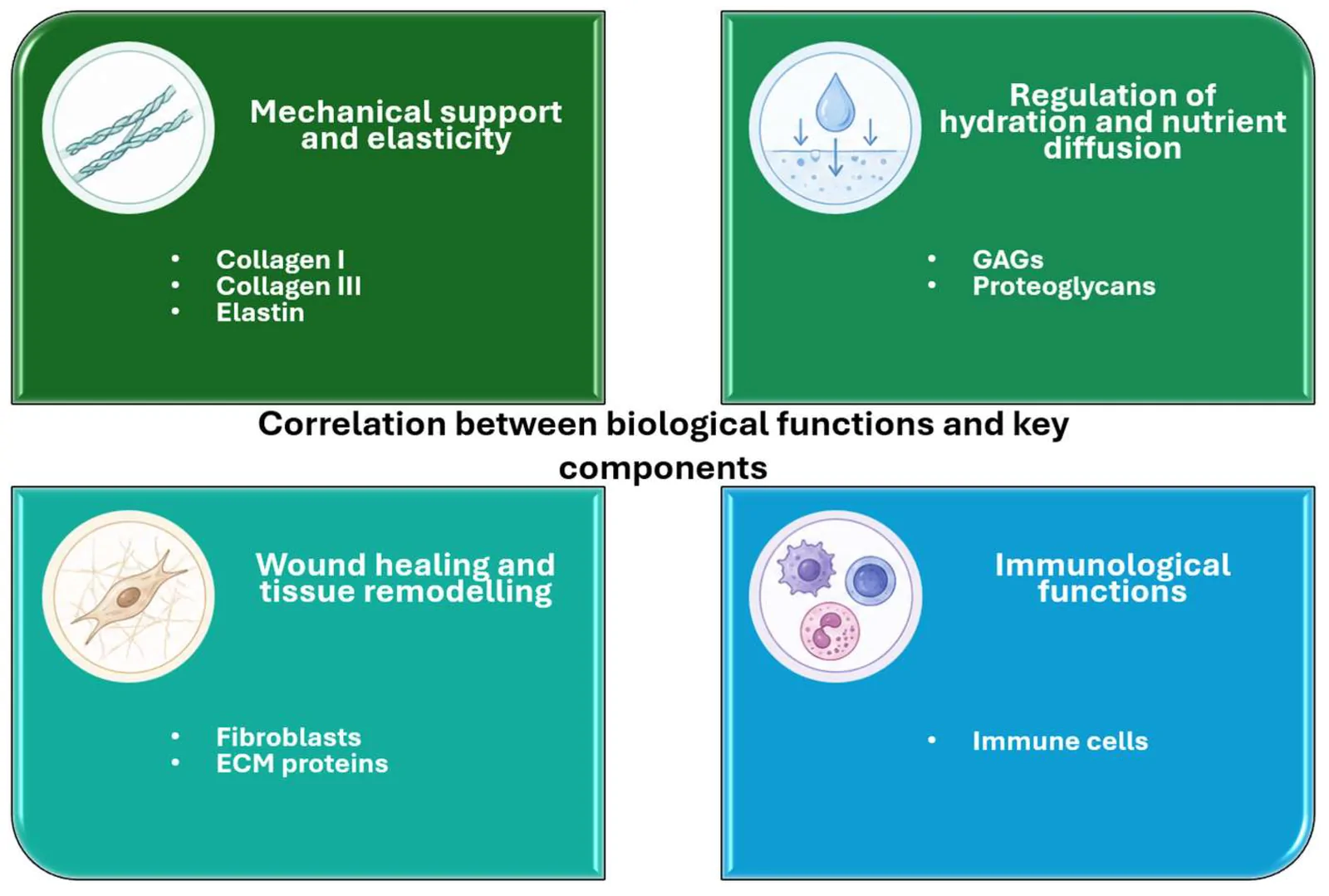
At-a-glance visual summary of the correlation between biological functions and key components. Created based on information from [36].

The dermis provides structural support and serves as a substrate for the epidermis. It is a connective tissue layer, and its main cell residents are fibroblasts, which have the crucial role of producing and maintaining the extracellular matrix (ECM). Moreover, it is composed of interstitial components (collagen fibers, elastic tissue, ground substance), cellular components (fibroblasts, mast cells, plasma cells, lymphocytes, dermal dendritic cells, histiocytes), blood vessels, lymphatic channels, and sensory nerves [33]. This ECM is mainly composed of collagen (type I predominantly—around 80%, and type III—around 10%) and elastin fibers, which are dispersed within a proteoglycan and glycosaminoglycans (GAGs) containing ground substance of non-fibrous nature. The increased number of glycans ensures structural integrity, hydration, orientation, and protection against proteolysis, as well as the sequestration of growth factors and cytokines. The mechanical integrity of the dermis is regulated by short crosslinking cell-surface molecules. All these components serve as stabilizers for the ECM, as well as an at-the-ready army of inactive enzymes, growth factors, cytokines, and structural molecules awaiting activation to take part in tissue repair or wound healing when needed [34]. A correlation between these key components and their biological functions can be seen in Figure 3. The skin appendages mentioned at the beginning of this section (hair follicles, sebaceous/sweat glands) also reside here, as well as cutaneous nervous and vascular tissues [29].

In contrast to the epidermis, the dermis is only composed of two layers: (i) the papillary dermis—superficial layer amounting to highly vascular loose connective tissue, residing deep into the epidermis; and (ii) the reticular layer—deep layer defined by an interlacing of collagen fibrils that constitutes the bulk of the dermis [34,35]. Together, these structural features provide a basis for understanding the importance of each component and their specific roles: the highly organized collagen network is in charge of strength and structural integrity, the elastin fiber system provides elasticity, and the glycosaminoglycan-rich ground substance regulates hydration.

As mentioned earlier, apart from the interstitial components, the dermis is also composed of cellular components. Table 2 provides a detailed overview of the resident cells in the dermis, alongside their characteristics and functions.

It is noteworthy that the dermal ECM is not just a structural scaffold; it also acts as a signaling platform that influences cellular behavior and coordinates tissue regeneration through integrin-mediated signaling and growth factor sequestration [49]. When discussing the regeneration capacities of tissue, the immune system plays a critical part. Wound healing starts with a local immune response that involves the recruitment of immune cells, which produce pro-inflammatory cytokines (TNF-α, IFN-γ). In addition, immune cells secrete growth factors that initiate the subsequent proliferative phase of healing. Moreover, triggering TGF-β1 promotes the formation of vascularized granulation tissue characterized by fibroblast–myofibroblast differentiation and the deposition of interim ECM that leads to the fibrous scar-converting tissue. As a last step, the dermal matrix is remodeled, and simultaneously, the epidermal tissue is reconstituted, resulting in wound closure. Following an injury, the activation of the immune response is critical and determines the outcome of tissue regeneration. Immediately after injury, the activity of specific cells marks the initial, immediate defense against infection (neutrophils, innate lymphoid cells, T helper 1 cells (T_H_1 cells), and M1 macrophages). Nevertheless, if this response is not appropriately regulated, it can lead to chronic inflammation, which impairs tissue regeneration and can cause further damage. Secretion of anti-inflammatory cytokines and growth factors falls under type 2 immunity, which implies the involvement of type 2 innate lymphoid cells, T helper 2 cells (T_H_2 cells), and M2 macrophages. This aims to generate a regenerative microenvironment that supports efficient cell replacement and restores tissue functionality and architecture, which has been reported to be linked to fibrosis. Among the different immune cells, restoration of tissue homeostasis has been strongly linked to macrophages and their phenotypes. M1 macrophages play a main role in recruiting immune components against pathogens, removing cellular debris, and initiating angiogenesis. Vital in wound repair are also dominant interleukin-4 (IL4)-activated M2 macrophages, which can promote ECM remodeling and synthesize several cytokines and growth factors (e.g., EGF, VEGF α). However, as previously mentioned, improper downregulation of M1 activation can lead to fibrosis. Therefore, wound healing is strongly connected to the regulation of the immune response and can be used as an active therapeutic target to control healing quality, improve tissue regeneration, and minimize scar formation [50].

Based on all of the above, it is becoming clear that the implications for biomimetic scaffold design must take into account the structural and functional complexity of the dermis. From architecture, composition, and mechanical behavior to enabling vascularization, immunomodulation, and dynamic tissue remodeling, these factors are key to transitioning from passive wound coverage to functional dermal regeneration.

## 3. Principles of Biomimetic Dermal Scaffold Engineering

In the 1950s, for the first time, the term ‘biomemetic’ was used by Otto Schmitt, and 20 years later, it made its public debut in Webster’s Dictionary. Biomimicry in tissue engineering and regenerative medicine refers to efforts to imitate the native microenvironment, from morphology to physiological conditions [51,52]. When designing a tissue-engineered dermal substitute, there are several interconnected principles that need to be taken into consideration so that the scaffold can efficiently mirror the structure, function, and biological activity of the native dermis, as compared to early-generation wound dressings, where the main role was to act as a passive protective barrier; modern biomimetic scaffolds are designed to be dynamic and actively participate in the regenerative process. Therefore, there is a need for understanding the complex microenvironment of the dermis (which was covered in the previous section), as well as the engineering behind the characteristics a material requires to translate into biological outcomes. Apart from physical and mechanical properties, biomimetic dermal scaffolds must also exhibit appropriate topography and microstructure to promote cell adhesion, proliferation, and differentiation [53,54,55].

Meeting all these criteria simultaneously requires careful optimization across several engineering parameters. The principles outlined in this section—scaffold architecture and porosity, mechanical properties and structural stability, biodegradation and tissue remodeling, bioactivity and cell–matrix interactions, and angiogenic potential—represent the five main values that determine the evaluation and advancement of biomimetic dermal scaffold design. It is worth noting that the presented principles are not independent; for example, the pore geometry will influence mechanical performance, while the architecture will affect angiogenic capacity, as they are all interconnected. The following subsections will examine each principle in turn, based on recent experiments and clinical evidence that emphasize both the state of the art in the field and its remaining challenges.

### 3.1. Scaffold Architecture and Porosity

One of the fundamental design parameters for a dermal scaffold is the three-dimensional (3D) architecture because it affects cell interaction and migration throughout. In addition, the porosity of the scaffold influences nutrient transfer, vascularization, and the development of functional tissues. Therefore, parameters to consider for optimal tissue formation include material composition, morphology, mechanical properties, and biocompatibility [56]. Pore size must be carefully controlled for its specific biological function; for example, pores that fall between 1 and 2 µm promote cell attachment in the epidermis, and by increasing the size up to 12 µm, the migration of dermal fibroblasts is aided, and larger pores (40–100 µm) promote vasculogenesis [56]. Pore interconnectivity is equally important because it promotes nutrient diffusion and waste removal. It has been noted that scaffolds with 60–90% porosity are appropriate for wound-healing applications, since they provide ample space for cell activity, nutrient and oxygen exchange, and new ECM production. While high-porosity scaffolds have demonstrated efficacy in supporting cell migration and infiltration throughout the structure, higher porosity results in lower mechanical strength; therefore, the balance between the two parameters is a crucial design consideration [57]. A promising approach would be to design multilayer scaffolds with different pore sizes that match the structural properties of distinct skin layers [58]. For example, Zandi et al. developed a bi-layered laponite-doped scaffold using GelMa hydrogel (12 µm pore size layer) and electrospun gelatin nanofibers (2 µm pore size layer)—interconnected structure—which facilitated nutrient transport while blocking unwanted cell infiltration [59]. A valuable study by Liu et al. determined the pore diameter of normal human dermis (219.29 ± 34.27 µm) by using continuous serial tissue sectioning to reconstruct a 3D model of native dermis. This provided a standardized anatomical dataset for the design of biomimetic tissue-engineered scaffolds and established that pore size must exceed fibroblast diameter [60].

### 3.2. Mechanical Properties and Structural Stability

The mechanical properties not only influence the integrity of the scaffold, but also actively regulate cell behavior, differentiation, and ECM deposition. As a result, they must mimic the mechanical features of the native dermis to evoke the same cellular response. For example, a 2018 study found that scaffolds made from electrospun porous nanofibers can replicate the ECM structure of the skin, with increased surface area for cellular attachment and for nutrient and oxygen penetration. Moreover, to replicate the elastomeric behavior with enhanced biocompatibility, a blend of polymers with combined properties was reported as the scaffold to overcome their individual drawbacks [54,61]. Another mechanical feature that influences cellular response, particularly fibroblast behavior, is scaffold stiffness. It has been noted that collagen matrices with varying collagen density and stiffness induce distinct cellular responses, notably higher proliferation rates with increased stiffness [62]. Thus, determining the proper stiffness of a dermal substitute is not only a structural feature, but also determines biological responses. Achieving the right balance between mechanical integrity and porosity remains an engineering challenge.

The use of synthetic polymers (such as polylactic acid (PLA) or polycaprolactone (PCL)) has received considerable attention due to their ease of processing and controllable mechanical properties and degradation rates, which allow precise control over the degree of hydrophilicity, stiffness, and degradation kinetics of the scaffold. Moreover, to enhance bioactivity, studies have focused on either altering the polymer architecture or using surface engineering approaches, such as coating, grafting, or plasma treatments. The use of composite biomaterials (both synthetic and natural) can overcome the limitations of individual components and combine the advantages to achieve tailor-made scaffolds with the desired physico-chemical and biological properties [55].

### 3.3. Biodegradation and Tissue Remodeling

Scaffolds used for skin tissue regeneration should provide mechanical support and cell adhesion sites, as well as a degradation rate that is aligned with skin regeneration. In this regard, natural fibrous protein biomaterials (collagen, silk proteins, elastin) have been extensively used for such applications, precisely for their excellent biocompatibility and biodegradability [63]. ECM-based biomaterials are degraded by enzymatic pathways that are associated with metalloproteinases (MMPs), collagenases, and other enzymes that target collagen, elastin, and GAGs. This is a highly regulated remodeling process that mimics native tissue restoration and wound-healing mechanisms. In addition, the by-products of the degradation process are not only biocompatible but can also promote regeneration through bioactive peptide release. These bioactive peptides will further encourage cellular activity, angiogenesis, and matrix synthesis. However, a specific balance needs to be maintained during the degradation process. If the degradation rate is too high, it can compromise scaffold integrity and tissue formation. In contrast, a slower-than-normal rate can prevent tissue remodeling and integration. An example is the use of synthetic polymers, which degrade by hydrolysis of the ester bonds in their backbone. This process is both predictable and adjustable, providing a critical advantage for coordinating scaffold degradation with tissue formation. Nevertheless, a disadvantage is that hydrolysis generates acidic by-products that can induce inflammation or cytotoxicity if not properly managed [55]. The Dermal Regeneration Template (DRT) has proven to be a well-established clinical standard. It is a collagen-GAG scaffold optimized to generate bioactive ECM and has been clinically demonstrated in both animal experiments and burned patients. Its residence time falls between 5 and 15 days, providing sufficient time to support dermal regeneration while being remodeled continuously by migrating fibroblasts and endothelial cells [64,65].

### 3.4. Bioactivity and Cell–Matrix Interaction

As mentioned earlier, an important feature of a biomimetic dermal scaffold is not only to provide structural support, but also to promote cell activity. This bioactivity stems from signals provided by GAGs, growth factors, and ligands incorporated into the scaffold, which interact with cell receptors and promote proliferation, differentiation, and ECM synthesis [55]. Although GAGs have shown increased bioactivity, native GAGs lack precision. Therefore, tuning GAGs could enable enhanced biological performance and integration in tissue-mimicking platforms. Apart from hyaluronic acid (HA), all GAGs naturally present sulfate groups. It has been noted that, due to their stable negative charge conferred by sulfation, GAGs bind to several proteins, chemokines, and growth factors, and they have a distinctly strong interaction with transforming growth factor-beta 1 (TGF-β1) and bone morphogenetic proteins (BMPs). Moreover, the degree of sulfation also influences fibroblast adhesion to ECM-like scaffolds [66,67]. By tailoring natural biomolecules, such as GAGs, advancements can be attained for next-generation materials to improve biomimicity of tissue-engineered scaffolds.

Bioactivity and cell–matrix interactions in a biomimetic dermal scaffold are not isolated properties, but result from the interplay of the previous design principles. The porosity of the scaffold has a key role in replicating the complex ECM, which directly affects the integration and migration of cells, transfer of nutrients and oxygen, and formation of functional tissue [57]—and it is this microenvironment that determines how effectively bioactive the scaffold is. Similarly, mechanical features directly affect bioactivity by inducing cellular responses based on ECM stiffness; the same occurs for biodegradation, which amplifies bioactivity through degradation by-products.

### 3.5. Angiogenic Potential of Biomimetic Scaffolds

One of the key factors, and arguably the most challenging one, in dermal scaffold engineering is vascularization, because without proper blood supply, the scaffold would be inefficient in sustaining cell viability or integration. Prevascularization is one of the main vascularization approaches in tissue engineering. To enhance vascularization, scaffolds can be prevascularized to contain a vascular network that connects with the host’s blood vessels and promotes cell survival, differentiation, and scaffold integration, a process known as inosculation. This can be attained by growing endothelial cells (ECs) in vitro within the dermal scaffold. There are several requirements that need to be considered when developing prevascularized scaffolds: (i) easy to harvest, (ii) low risk for the patient, (iii) high proliferation in vitro, and (iv) low immunogenic potential. One of the most frequently used ECs for this application is human umbilical vein ECs (HUVECs) due to their ease in extraction and culture preservation and their low immunogenicity. It has been noted that the use of ECs alone may present several limitations and can lead to incomplete vascular structures. Therefore, co-culture systems with supporting cells are a known strategy for promoting vascularization [68]. Amongst these supporting cells, several studies have demonstrated the efficiency of fibroblasts, highlighting their ability to deposit a matrix that improves the mechanical properties of the ECM, facilitating cell migration and proliferation [69,70]. During the angiogenesis process, complex signaling takes place between cells (fibroblasts, endothelial cells) that promote the activity of growth factors, such as vascular endothelial growth factor (VEGF). This specific growth factor is vital during wound healing and repair because it promotes angiogenesis and vascularization, respectively. It has been shown that VEGF-activated scaffolds with transfected dermal fibroblasts and endothelial cells exhibited enhanced VEGF expression and endothelial cell migration, as well as vascular-like structure organization [71]. Even though adding angiogenic factors to dermal substitutes is a powerful approach to induce angiogenesis, it presents several disadvantages—apart from the high cost of these angiogenic factors, it has been noted that they are unstable, with a short lifespan, and are prone to inactivation. As an alternative, gene-activating scaffolds have been recently investigated. Weng et al. developed a dual gene-activated dermal scaffold that contained multiple plasmic DNAs encoding VEGF and angiopoietin-1 (Ang-1). The results highlighted enhanced cell infiltration, collagen deposition, blood vessel formation, and vascular maturation. Moreover, compared with single-gene-activated scaffolds, dual-gene scaffolds show greater potential for augmented angiogenesis [72]. Another strategy is radially aligned nanofiber scaffolds incorporating dual growth factors. Fan et al. developed such a scaffold incorporating epidermal growth factor (EGF) for epidermal cell proliferation and differentiation, and VEGF to stimulate angiogenesis. It was noted that the resulting scaffold not only stimulates rapid healing but also enhances the quality of the remodeled tissue [73]. Another critical aspect is the passive diffusion of oxygen and nutrients through the tissue-engineered scaffold. If the fabricated tissue is thicker than 2 mm, the angiogenesis process will require a longer time to be completed. Complete vascularization of an engineered dermis can take up to two weeks, and during this time, the grafted tissue survives through the diffusion of oxygen and nutrients from the host. However, this is limited to a distance of 100–200 µm [74,75]. Because of this diffusion limit, the cells present in the core of a thicker scaffold are not likely to receive the proper nutrients and oxygen to sustain themselves, thus compromising vascularization and potentially leading to necrosis and implant failure [76]. All the previously discussed approaches (prevascularization, growth factor incorporation) can aid the development of vascularization and the integration of the engineered scaffold. Needless to say, the angiogenic potential is also affected by the architecture and pore size of the scaffold, as discussed previously, and combining this with the integration of growth factors or genes provides a promising strategy for developing biomimetic dermal substitutes with angiogenic potential.

## 4. Classes of Dermal Substitutes and Clinical Applications

Available dermal substitutes are broadly classified based on their composition and origin into five categories: acellular, cellular, xenogeneic, synthetic, and hybrid. Each class has distinct structural and biological properties that lead to specific clinical advantages and limitations. Table 3 summarizes these characteristics alongside representative examples and their key distinctions. This classification can sometimes overlap in practice, with several products classified into multiple categories simultaneously. No single classification can fully eliminate overlap given the diversity of the scaffolds and their potential applications.

Dermal substitutes have evolved from simple wound-care dressings to complex reconstructive scaffolds used across a wide range of clinical applications. Even though there are several classes of dermal substitutes, they all share a common functional principle: to provide a scaffold (cellular or acellular) that supports angiogenesis, cell activity, and new tissue formation. In the following section, the clinical outcomes of different dermal substitutes will be addressed for several applications: burn reconstruction, traumatic soft tissue defects, oncologic reconstruction, and chronic wounds and complex skin defects. It is worth mentioning that even though these substitutes share the above-mentioned common functional principle, each application comes with specific challenges and needs. Therefore, the choice between different substitutes will vary based on the different biological and clinical requirements.

Burn reconstruction is one of the main clinical applications that make use of dermal substitutes; however, it is challenging, especially in reconstructive tissue engineering, where donor sites are limited [116]. Burns that cover over 40% of the Total Body Surface Area (TBSA) are a clinical challenge due to the ratio between the wound area and available donor site, which leads to donor site morbidity. Dermal substitutes can reduce the thickness of split-thickness skin grafts needed to close the wound [117]. In deep and full-thickness burns, the dermal architecture is destroyed. During the healing process, there is high myofibroblast activity (a key role in wound contraction), and healing without a supporting scaffold can lead to hypertrophic scar tissue [118]. A major complication in burn wounds is infections resulting from wound contamination, induced immunosuppression, extended hospitalization, and invasive interventions [119]. In clinical practice, debridement is an important step in preparing the wound bed; however, it is sometimes insufficient to eliminate bioburden completely. While antimicrobial dressings showed promising results, they come with several disadvantages: they provide temporary protection, are limited in availability, and require repeated changes, thereby disrupting the healing process and increasing infection risk. Considering these factors, a dermal substitute must act as a multifunctional platform serving several purposed: protection, matrix for tissue regeneration, reduce infection and enhance immune response to prevent infection [120]. A meta-analysis study based on 31 comparative trials systematically reviewed the use of dermal substitutes in the reconstruction of burn scars and acute burns. The study concluded that although there was an initially slower wound-healing rate with dermal substitutes compared with conventional split-thickness skin grafts (STSGs), scar quality was enhanced; the results indicated minimal differences between the targeted groups. However, when assessing re-epithelialization, Matriderm showed a significantly lower post-surgery re-epithelialization rate than STSG. TransCyte exhibited a 7.5-day re-epithelialization period, the fastest compared with other cellular substitutes and conventional procedures. Moreover, dermal substitute groups reported overall lower values than control groups regarding graft take, regrafting, and healing time. For scar quality, when using acellular dermal substitutes, the resulting scar was observed to present normal dermal characteristics based on vascularity, pigmentation, pliability, and height, in contrast to STSG. Scar contraction studies between MatriDerm, Integra and STSG suggested that two-step Matriderm use may induce more contraction than Integra, but it showed that in one-step procedures it would potentially be more advantageous. However, further research is required [121]. After dermal scaffold application and the formation of new tissue, there is a window of opportunity for infection, which presents another clinical complication. By investigating the incidence of infection on patients treated with dermal substitutes and conventional procedures, a meta-analysis study concluded that there are no significant differences between the two based on nine randomised clinical trials (RCTs) and seven observational studies [122]. Amongst various dermal substitutes used in 28 RCTs for acute burn injuries, TransCyte presented the lowest infection rate and Matriderm with STSG (as compared to STSG alone) reported no infection at all [123]. Among the various dermal substitutes, one-stage application scaffolds represent a significant advancement, even though they come with stricter wound bed requirements. Currently, there is no ideal dermal substitute that can completely replicate the complex structure and functionality of native human skin. However, among the available resources, Integra remains the most commonly used scaffold for full-thickness burn reconstruction. The choice between a two-stage procedure—required by Integra—and a single-stage one—such as MatriDerm –depends on several factors, including wound state and available donor tissue. Synthetic matrices, such as Biobrane or Suprathel, are generally recommended for partial-thickness wounds where the main objective is scaffold integration and rapid re-epithelialization [124]. Even though advancements in dermal substitutes involve living fibroblast-seeded meshes (an example would be TransCyte—which could be seen as a synthetic Biobrane with added fibroblasts), these scaffolds are generally associated with high costs. In the case of the previous example, TransCyte is reported to be 15 times more expensive than Biobrane. Other examples include AlloDerm and Integra that cost 15–30 USD/cm^2^, and the cost can even double for fibroblast-seeded scaffolds, even four times for Apligraf; while keratinocyte-seeded scaffolds present even higher costs [125].

Traumatic soft tissue defects, which include damage or loss of skin, muscles, and tendons, present distinct requirements from burns. Due to differences in wound environment, the requirements for traumatic soft tissue defects differ from burn reconstructions. The main challenges include contaminated wound beds, exposed avascular structures (e.g., tendons, bones), and site-specific functional requirements (e.g., the hand). Negative pressure wound therapy (NPWT) has generally become a complementary technique in such cases. By maintaining a sealed environment, NPWT uses a vacuum pump to provide negative pressure to the wound bed. This therapy promotes granulation tissue formation while reducing edema and bacterial load [126]. Complex wounds favor two-stage protocols, such as Integra, whereas clean wound beds with smaller defects prefer one-stage procedures (MatriDerm). A main contribution of dermal substitutes in this area is their ability to minimize surgical morbidity, especially in cases where skin grafts are not suitable [127]. Compared with burn reconstruction, the scientific evidence for dermal substitutes in traumatic soft tissue defects is limited, with no RCTs comparing the five main categories (acellular, cellular, xenogeneic, synthetic, and hybrid) against each other. A study on long-term healing results in traumatic soft tissue defects of the lower limbs assessed healing rates and scar quality by comparing the use of STSG with (or without) MatriDerm. The study included 147 cases and found that scar tissue quality did not differ significantly between the two groups; however, it was noted that the addition of MatriDerm aided in bridging the exposed tissue, with a graft take rate of 60%. Moreover, preconditioning the wound bed with NPWT was shown to significantly reduce infection risk and enhance graft take rate [128]. A similar result was attained in a cohort study on full-thickness skin grafts. A combination of MatriDerm and STSG was used in traumatic lower limb soft tissue defects with graft take rates reaching 90%, including complex wounds after open fractures [126]. Another cohort study on 51 patients with complex upper-extremity wounds achieved a 92% wound closure rate by using a synthetic dermal substitute. Moreover, almost 40% of these did not require secondary skin grafting [129]. For severe upper extremity wounds with exposed bone or tendon, dermal substitutes are extensively used due to their ability to restore function and provide aesthetically pleasing results. Currently, Integra is widely regarded for such applications; however, reports of high cost and infection rates have restricted its use. Therefore, synthetic dermal substitutes have gained more attention with satisfactory clinical outcomes, lower infection rates, and lower direct costs [130]. A meta-analysis and systematic review of the efficiency of synthetic dermal substitutes for upper limb reconstruction highlighted a successful scaffold integration rate of 92%, a 95% graft take rate, and an 8.5% infection rate (lower than infection rates reported for Integra). The average integration time was 37 days, comparably longer than Integra (21–33 days) and MatriDerm (21–28 days). In the 12 studies reviewed, the use of synthetic dermal substitutes led to successful wound healing in 90% of the presented wounds [131]. Although these are significant findings, there is a need for future research on long-term scar and contracture outcomes, with RCTs that compare several categories of dermal substitutes for this specific application, with the current literature focusing on either synthetic dermal substitutes or acellular ones (MatriDerm).

Oncologic reconstruction can be particularly challenging after tumor tissue resection due to several factors: (i) the degree of tumor tissue removal, (ii) the need to preserve surgical margin orientation, and (iii) the often required supplementary radiation therapy, which compromises vascularization and healing capacity. Both Integra and MatriDerm have been reportedly used in oncologic reconstruction with similar outcomes [132]. Among the benefits presented for the use of dermal substitutes, the following are mentioned in the literature: reduced operating times, minimized donor site morbidity, and temporizing the wound bed while awaiting confirmation of clear margin resection. These benefits are highlighted in a comparison study between the use of Integra and MatriDerm for the reconstruction of cutaneous malignancies [133]. Integra was tested on 17 patients with soft tissue tumors, with a 100% graft take in 16 of the patients, and 97% graft take for the 17th. It was also noted that Integra has excellent results in patients with large defects, with reduced donor-site morbidity and exceptional outcomes regarding functionality [134]. A similar result regarding graft take was attained in a study on the use of Integra on complex scalp reconstruction following the excision of cutaneous dermal tumors. This large case study (101 patients) focused on the use of Integra in a single-stage procedure on both partial and full-thickness defects, with a success rate of 90%. The main complication post-operatively noted was infection in almost 21% of the cases; however, it was managed using antimicrobials. Post-operative infections were correlated with patients taking anticoagulants, with an overall complication rate of 30% [135]. An even larger study based on a systematic review of 40 articles, encompassing 898 patients who underwent Mohs micrographic surgery (MMS) and received dermal substitutes, revealed that the most common substitutes used were xenografts. One of the reasons presented is the affordability of these grafts, which are around $10 per cm^2^. Similar to traumatic soft tissue defects, it is highlighted that there is a lack of data comparing the different categories of dermal substitutes [136]. Another systematic review analyzing 40 studies, with a total of 687 patients, confirmed previous results in substitute selection. The main dermal substitutes identified were xenografts (porcine- and bovine-derived), with the main factors influencing selection being cost, healing efficiency, usability, patient comfort, and cosmetic outcome [137].

An important factor in dermal substitute selection for oncologic reconstruction involving areas such as the face, scalp, and hands is scar quality and contracture, since aesthetic and functional outcomes are critical. A study on the use of Integra for facial soft tissue reconstruction in patients who underwent MMS on the face highlighted successful reconstruction, with re-epithelialization of 90% of defects. The limitations of the use are also mentioned—high cost and its use on larger defects (>27 cm^2^); however, within the presented study, successful reconstruction was attained with aesthetic and functional outcomes, and a low complication rate [138]. When tumor resection takes place in acral surfaces, such as hands and feet, one of the main challenges is preserving functionality. Conventional surgical procedures, such as full-thickness skin grafts and secondary intention healing, come with limitations including scar contraction and lack of donor site for large defects. Despite the high cost and two-stage procedures, acellular dermal substitutes are preferred in such cases due to their exceptional functional and cosmetic outcomes [139].

Chronic wounds and complex skin defects, which are characterized by wounds that do not undergo the natural healing process within a desired timeframe, represent the largest and most economically significant application of dermal substitutes [140,141]. As compared to the previous applications, chronic wounds represent the most biologically hostile environment. A health assessment based on 40 studies concluded that using dermal substitutes in addition to standard care increases effectiveness in complete wound healing for chronic DFUs and venous leg ulcers (VLUs), compared with standard care alone [142]. Similar results were highlighted in a meta-analysis on studies that evaluated wound closure rate by 12 weeks. The systematic review included 25 studies and determined that dermal substitutes are 1.67 times more likely to heal in this time period as compared to standard-of-care dressings. Moreover, by reducing the time period by half, the likelihood of wound closure for dermal substitutes increased to 2.81 [143]. Some of the clinical challenges include a hostile wound environment—elevated MMP activity can degrade the applied scaffold before the regenerative effect can take place, high infection risk, poor vascularization, and comorbidities (e.g., diabetes). One of the most studied dermal substitutes for DFUs and VLUs with encouraging RTC evidence for both is Apligraf, formerly named Graftskin. A randomized prospective trial tested the use of Apligraf against a control group for the treatment of non-infected neuropathic DFU. It was noted that after 12 weeks, 56% of the patients treated with Apligraf achieved complete wound healing, as compared to the control group (38%) [89]. These results were in accordance with a meta-analysis of 14 RCTs on a total of 1524 patients. The study investigated comparative wound-healing outcomes in DFUs from patients treated with standard of care alone versus patients treated with standard of care and dermal substitutes. The group treated with dermal substitutes achieved higher complete healing rates with lower amputation risks; however, there were no significant differences between the two groups with regard to complication risks or recurrence rate [144]. A direct comparison of cellular (Apligraf, Dermagraft) and acellular (EpiFix) substitutes for wound closure rates of DFUs indicated that EpiFix had the highest percentage (92%) within 12 weeks. Moreover, the recurrence rate of EpiFix was 5.6% after 9 to 12 months, whereas Apligraf and Dermagraft reported 5.9% and 18.8%, respectively [145]. A substitute that addresses the MMP-degradation issue is Promogran (a collagen-based scaffold); however, its acellular composition does not provide growth factor delivery and is regarded more as a wound bed dressing rather than a tissue-engineered dermal substitute [85]. Infection is a critical factor that can influence the performance of dermal substitutes in chronic wounds and complex skin defects. A Technical Brief from 2020 described 76 commercially available dermal substitutes for the treatment of chronic wounds across 22 RCTs and three systematic reviews. However, it was highlighted that these studies rarely reported wound recurrence, infection, functionality as clinical outcomes, resulting in a significant evidence gap [146]. Where reported, it has been noted that dermal substitutes have lower wound-related infections compared to the standard of care, and no significant adverse effects have been associated with their use [147]. Notably, in 2023 the International Working Group on the Diabetic Foot (IWGDF) updated their clinical guidelines and issued a recommendation against using cellular and acellular skin substitutes as routine adjunct therapy for standard of care in DFUs. The rationale was based on 10 RCTs for cellular dermal substitutes and 13 RCTs for acellular dermal substitutes and included insufficient evidence regarding which dermal substitute is most efficient, cost-effectiveness, studies with high risk of bias, and limited data associated with reduction in amputation rates [148].

For better clarity, the biological and clinical differences between the above-described dermal substitutes have been summarized in Table 4.

## 5. 3D Bioprinting

A future perspective in the field of dermal substitutes currently undergoing advancements that require special attention is 3D bioprinting. This technique has the potential to overcome the limitations presented by commercially available skin substitutes, such as a lack of skin appendages, vasculature, and innervation, and provide personalized scaffolds [155].

**Figure 4:**
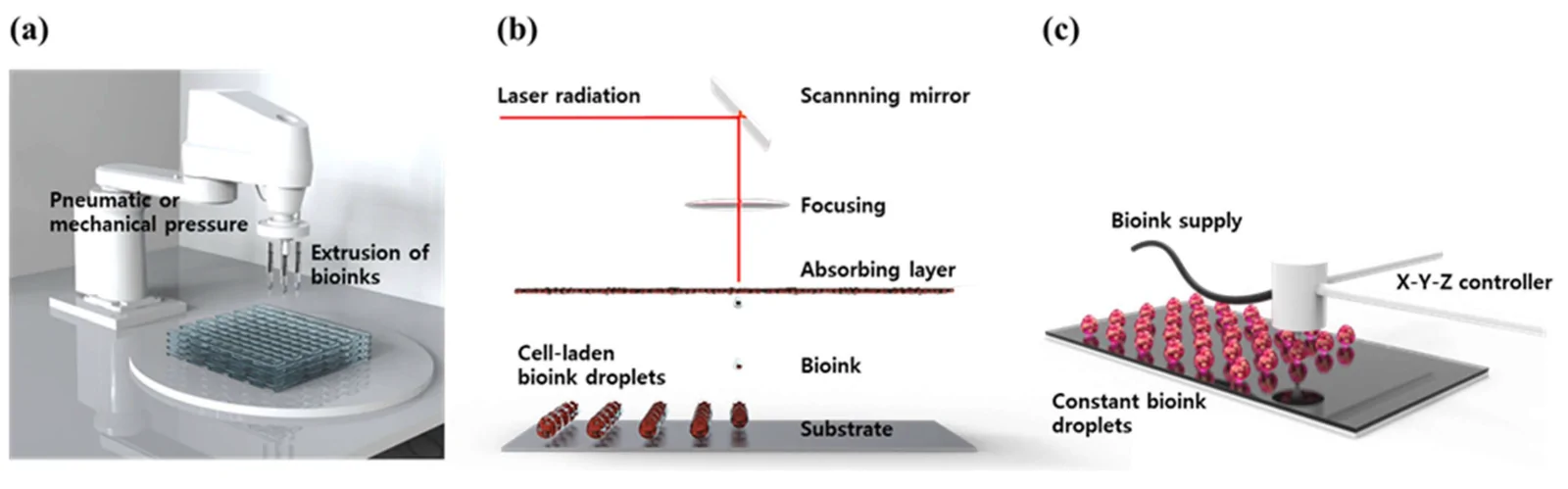
3D bioprinting methods: (**a**) extrusion-based bioprinting, (**b**) laser-assisted bioprinting, and (**c**) droplet-based bioprinting. Reprinted from Ref. [156].

3D bioprinting is a highly reproducible process that allows the fabrication of controlled structures through a layer-by-layer building process. The current focus in the skin regeneration sector is on three novel technologies: extrusion-based bioprinting (EBB), laser-assisted bioprinting (LAB), and droplet-based bioprinting (DBB) (Figure 4) [156,157]. The EBB technique is based on the following concept: bioink (a mix of biomaterials and living cells) is extruded through a micro-needle and deposited layer-by-layer on a platform to produce the scaffold (a 3D wound shape that matches the patient). This technique allows personalization, live cell processing, as well as replication of the complex architecture of the skin. Some of the limitations of this technology include poor bioprinting resolution, cell damage, limited mass-production, and biomaterial selection [158,159]. The LAB system uses laser light focused on a metal film deposited on the back of silicate glass, leading to the evaporation of the biokin, turning it into droplets that are sprayed onto the substrate. This non-contact printing technique offers excellent printing resolution compared to EBB and high cell survival rates; However, the main disadvantages are slow printing speed and comparatively higher costs than other techniques [160]. DBB technology includes Inkjet bioprinting (further classified into continuous inkjet and drop-on-demand printing) and Electrohydrodynamic jetting bioprinting. They use a mix of hydrogels containing living cells, which are dispensed in the form of micro-droplets to form multi-layer scaffolds that replicate the structure of the skin. The main advantage of these techniques is that they can precisely deposit small droplets of bioink in a non-contact fashion. However, the challenges include limited biomaterial selection, potential nozzle clogging, and cell stress [161]. A disadvantage that comes across all the presented bioprinting techniques is that the resulting product, with a specific form, is constructed on a flat surface and therefore can lead to a misfit of the construct on the defect area. In situ bioprinting, however, offers the possibility to deposit the bioink directly inside the defect in vivo and has been presented as an alternative strategy that can translate bioprinting to clinical practice [157]. A review paper by Smandari et al. addresses the development of these strategies, advantages, disadvantages, and future improvements [162].

Despite the different types of bioprinting techniques, an essential component for all of them is the bioink. An ideal bioink should have the desired physicochemical characteristics that result in: (i) a sufficient mechanical strength to generate the scaffold, but also match the mechanics of the skin tissue (preferably tunable), (ii) adjustable gelation and stabilization to replicate the desired shape, (iii) biocompatibility and/or biodegradability, (iv) it should allow chemical tailoring for tissue-specific needs, and (v) large-scale production with high reproductibility. Numerous natural and synthetic biomaterials have been investigated for their use as bioinks, including alginate, collagen, gelatin, decellularized matrices, bioinks based on growth factors, polyethylene glycol (PEG), polycaprolactone (PCL), cell aggregate-based bioinks, composite bioinks, and bioinks with bioactive molecules [163].

### 5.1. Preclinical Evidence

A 3D bioprinted protocol to produce skin tissue models with high cellular complexity was developed and showed the reproducibility of human vascularized skin tissue in multi-well plates. By making use of a commercially available bioprinter, the presented protocol introduced several novelty factors: the bioink contained neonatal fibroblasts, pericytes, and human induced pluripotent stem cell (iPSC)-derived ECs with a fibrin-based hydrogel, which was printed in a transwell format with a biodegradable poly(lactic-co-glycolic acid) (PLGA) scaffold to manufacture the vascularized dermis. Keratinocytes were added on day seven after bioprinting, and differentiation and epidermis maturation took place over 12 days. The resulting construct exhibited a layered structure consistent with the architecture of dermis and epidermis. The purpose was to develop skin tissue that can generate disease models of atopic dermatitis, which can provide a platform for testing potential drug treatments [164]. A preliminary study based on a pilot swine animal study for deep third-degree burns presented a surgery-ready robotic 3D bioprinter that could potentially offer a bioprinting approach for the treatment of burn injuries in vivo. It was highlighted that the use of cell-laden bioink had a direct correlation to skin regeneration, generating in situ fibroblast growth factor and VEGF, which led to the re-epithelialization of the wound [165]. Several murine models used as in vivo models have been used to evaluate the efficiency of 3D-printed bioinks for wound healing: human mesenchymal stem cells (MSCs)-containing matrices for enhanced regeneration in diabetic mice [166], multi-cellular constructs that can induce angiogenesis for vascularized skin regeneration [167], improved antioxidant activity of curcumin-loaded scaffolds for enhanced in vivo diabetic wound healing [168], proof-of-concept mobile skin bioprinting systems for the management of full-thickness wounds [169]. Moreover, a 3D-printed scaffold was fabricated from porcine skin ECM that can monitor oxygen through an integrated microsensor as a novel approach for diabetic ulcer treatment. In vitro and in vivo studies highlighted the cytocompatibility and low immunogenicity of the scaffold with the possibility to tailor it per patient requirements [170].

### 5.2. Clinical Evidence

The current literature focuses on the potential clinical use of 3D bioprinted scaffolds for dermal substitutes; however, little to no evidence from clinical trials conducted in humans has been reported. The most clinically advanced paper to date is based on a robotic 3D bioprinter used in situ to enhance tissue regeneration in a porcine model and a first-in-human trial. The results highlighted an increased wound closure rate with the 3D-printed construct compared with healing by secondary intention in the model. This result enabled the translation to a first-in-human clinical trial and provided a feasible, safe, and scalable approach for the delivery of autologous dermal scaffolds in situ [170].

## 6. Limitations of Current Evidence

Despite decades of studies and progress in the field of biomimetic dermal substitutes, there is currently no available product that can successfully fully replicate the anatomy and functionality of native dermal tissue. To date, skin substitutes, regardless of their class, have only partially addressed the requirements for complete wound closure. The main shortcomings include inadequate pigmentation (either hypo- or hyperpigmentation), lack of stable vascular and lymphatic networks, inadequate innervation, or epidermal appendages [150,171]. Apart from the mentioned limitation, skin substitutes also differ from regenerated tissue by exhibiting greater contracture and loss of sensitivity, leading to significant mobility and aesthetic concerns [93]. A particularly neglected structural gap in substitutes, especially for full-thickness wound healing applications, is the absence of the hypodermal layer. While there have been notable efforts to address this issue, it remains challenging and often requires the use of synthetic components to replicate the structural and functional properties of the subcutaneous adipose layer [27,172]. Another limitation is delayed vascularization, which can directly affect the success of the grafting procedure. Acellular dermal substitutes can take up to 2–4 weeks to achieve proper vascularization in order to support a split-thickness skin graft. However, a prolonged avascular phase (two weeks or longer) creates a hostile environment for cellular survival, thereby minimizing the desired effect and delaying wound closure [150]. This represents a significant clinical challenge, especially for patients with wounds that cover a larger surface area and cannot allow exposure for prolonged periods.

A 2025 white paper specifically addressing the legal considerations for autologous dermal substitutes in Europe highlighted that even though there have been considerable technological advancements in the field, there are still considerable obstacles, such as production cost, scalability, and long-term effectiveness, and emphasized the effect on production and availability of tissue dermal substitutes generated by regulatory changes [173]. The existing regulatory frameworks, both in Europe and the U.S., demand comparability requirements—a standard that hinders scale-out production. Moreover, cell-based product regulations mostly fall under conventional pharmaceutical regulations, which do not adequately account for the specific characteristics of these therapies (reduced lot sizes, short shelf-life, and donor material variability) [174].

Heterogeneity of clinical studies also represents a limitation in the field because it hinders cross-comparisons between studies. A 2025 meta-analysis on acellular dermal substitutes in pediatric burns concluded that results of the study should be carefully interpreted due to the high level of heterogeneity and methodological diversity between the analyzed studies and that the findings should be confirmed by further research, including standardization protocols and an expanded, more diverse patient cohort [175]. Moreover, the RCT data available generally compare dermal substitutes to standard care, and not each other. There is a need for additional RCT data comparing the various substitutes to achieve a proper understanding of which clinical setting they are best suited for [176]. The current available literature lacks meaningful long-term follow-up studies. These studies are essential for evaluating critical aspects such as scarring, contracture, or mechanical durability. Initial studies suggest that autologous dermal grafts have better early results, but tissue-engineered skin substitutes achieve comparable long-term outcomes with reduced donor-site morbidity [27].

The economic evidence provided for dermal substitutes for skin regeneration is scarce when not taken as individual categories. Currently, there are no cost-effective analyses comparing the different substitute classes, and the reported values presented are generally product-specific. Even though some products have a well-established price (e.g., Apligraf) and comparisons between specific products have been identified in the literature (e.g., TranCyte—Biobrane), there is no economic data regarding the cost-effectiveness of the various treatments.

## 7. Conclusions and Future Perspectives

The field of biomimetic dermal substitutes for skin reconstruction has seen significant advancements over the past decade, transitioning from traditional dressings to complex constructs that can almost replicate the architecture and functionality of native dermis. Regeneration techniques that support and enhance the dermal self-healing potential have gained considerable attention. However, the current pool of biomaterials used still faces challenges. Amongst these, accurately mimicking the properties and structure of natural skin and regulating the microenvironment are critical [177].

The five principles examined in this review, namely scaffold architecture and porosity, mechanical properties and structural stability, biodegradation and tissue remodeling, bioactivity and cell–matrix interactions, and angiogenic potential, all define the engineering backbone that helps tailor-make biomimetic dermal scaffolds. These principles are not independent but highly interconnected, influencing one another. The presented classes of dermal substitutes each have a specific clinical role, with advantages and disadvantages. There is no universal substitute for all wound types since the prioritized requirements for each application differ. Acellular and collagen-based scaffolds have gained popularity for their ability to support cellular integration and activity. While acellular scaffolds are closer to the ECM architecture, collagen-based matrices offer greater flexibility and control over degradation rates [110]. Xenogeneic substitutes are generally preferred for temporary wound coverage; however, because they are not a permanent option, their applications are restricted. Synthetic scaffolds present several advantages, including high manufacturing reproducibility and no risk of disease transmission; however, they lack inherent bioactivity and generally require surface modification for cellular support. By combining natural components with synthetic polymers, hybrid scaffolds show promise due to the combined advantages of the individual components; however, they are limited in clinical use due to high costs and limited availability.

The field of biomimetic dermal substitutes is slowly rising, but it still presents clinical challenges. The limitations presented in the current literature prevent definitive conclusions regarding their efficiency and hinder their clinical use. Continuous advancement in the field, alongside standardized trial protocols and uniform outcomes, is still required. 3D bioprinting of dermal tissue (including its appendages) is a major advancement, enabling reproducibility, accuracy, scalability, and the personalization of scaffolds with complex structures [178]. Recent studies focused on 3D bioprinting have highlighted several advantages of this technique, including biocompatibility and the production of cell-seeded scaffolds that closely mimic the complex architecture of native dermis (multilayered), thereby overcoming current limitations and providing a promising alternative [179].

Delayed or immature vascularization is an ongoing challenge for dermal substitutes. However, ongoing research has identified several engineering strategies to overcome this limitation: pore architecture optimization, growth factor or gene delivery, and fabrication of prevascularized scaffolds. A study on the fabrication of a prevascularized bioengineered dermis and its implantation in a mouse full-thickness skin defect model reported promising results: after the first week of implantation, capillaries and complete epithelialization were observed, and after two weeks, innervation and skin appendages developed [180]. Currently, commercially available dermal substitutes are biologically limited by the absence of skin appendages and sensory innervation. This is an area of the field still in progress, and although there has been significant progress, especially in mouse models, there is still a long way to go toward successful translation to human skin. An ideal dermal substitute should include all cutaneous adnexa and a functional vascular and nerve network to enhance the integration of the scaffold in the host tissue with minimal scarring [181]. The commercial trajectory of regenerative medicine is receiving promising external validation for scientific advancement. The global regenerative medicine market, valued at USD 43.77 billion in 2025, is projected to reach approximately USD 234.85 billion by 2035, with a Compound Annual Growth Rate (CAGR) of 18.29%. Moreover, biomaterials currently dominate the market share at 51%, which reflects a shift toward natural approaches [182].

Tremendous advancements have been made in the field to date; however, despite the significant research literature and the commercial trajectory, the field lacks comparative studies on different skin substitutes. RCTs and clinical trials present different protocols and follow-up spans, various outcomes, and few report patient outcomes, wound recurrence, or sensory recovery. Future trials should address these inconsistencies to provide cross-product comparisons that will facilitate the choice between them based on performance in specific applications.

Although significant translational challenges remain, continuous advancement in biomaterials, bioactive scaffold design, and emerging technologies such as 3D bioprinting leads to ongoing progress in the skin reconstruction field. Interdisciplinary collaboration and standardized clinical evaluation will be essential to translate these findings into reliable, patient-specific therapies capable of restoring not only tissue integrity, but also the complex biological functions of native skin.

## Figures and Tables

**Table 1:** Classification of complex skin defects.

Criteria	Examples	Importance for Reconstruction	References
Chronicity	Acute Chronic	Establishes whether immediate closure is required or wound bed preparation is needed.	[11,15]
Etiology	Burn Diabetic Infectious Pressure injuries Venous Trauma	Influences blood supply, inflammation, recurrence risk, and the reconstructive technique used.	[11]
Depth	Superficial Subcutaneous Composite defects	Extensive defects may require microvascular tissue transfer and may limit reconstruction.	[16,17]
Wound bed	Vascularized Compromised Non-graftable	Determines the outcome of the reconstruction.	[18,19]
Size, geometry, and site	From small to extensive From linear to 3D Site: face, extremities, etc.	Influences functional and aesthetic requirements	[16,17]
Wound assessment	Infected vs. clean Necrosis vs. healthy Erythema Wound edges	Determines the immediate steps, such as reconstruction timing	[15]

**Table 2:** Dermis cellular components.

Category	Type	Distribution	Primary Function	Refs.
Primary (structural)	Fibroblasts	Higher activity in the papillary than the reticular dermis	ECM synthesis and remodeling	[34]
	Myofibroblasts	Transient	Physiological reconstruction of connective tissue after injury, fibrosis	[37]
	Fibrocytes	Recruited from peripheral blood following injury	ECM synthesis and differentiation, wound healing, fibrosis	[38]
Immune	Mast cells	Near blood vessels, nerves, and hair follicles	Angiogenesis, homeostasis, innate/adaptive immunity, activation/mediator release	[39,40]
	Macrophages	Throughout the dermis	Homeostasis maintenance, phagocytosis, wound repair, and inflammation regulation	[41]
	Dendritic cells	Predominantly in the papillary dermis	Detection and processing of antigens (immature cells), immune system activation (mature cells)	[42,43]
	T lymphocytes	Throughout and clustered in perivascular areas	Immune sentinels	[44]
Vascular (supporting)	Endothelial cells	Throughout, they line blood/lymphatic vessels	Repair during wound healing, oxygen and nutrient delivery, waste/metabolic removal	[34]
	Pericytes	Predominantly in the papillary dermis	Permeability and structural stability regulators in blood vessels	[45]
	Glomus cells	Reticular dermis—predominantly in digits (fingertips and toes)	Thermoregulation	[46]
Neural-associated (supporting)	Schwann cells	Dermis—associated with unmyelinated nerve fibers	Repair and regeneration	[47]
Metabolic (supporting)	Adipocytes	Reticular dermis	Immunity, thermoregulation, hair growth regulation, energy storage, wound healing	[48]

**Table 3:** Dermal substitutes.

Class	Examples	Structural Characteristics	Biological Characteristics	Advantages	Disadvantages	Distinctions	Refs.
Acellular	Integra	Crosslinked bovine collagen + GAG layer with temporary silicone sheet; Pore size: 70–200 µm	Silicone layer—temporary epidermal barrier; Promotes fibroblast infiltration and angiogenesis; Biodegrades over 3–4 weeks—replaced by neodermis.	Widely reported for full-thickness burns; Readily available.	Two-stage procedure; Infection risk.	Maintains the native ECM architecture without cells. It is a two-stage procedure (first dermal layer, then epidermal autograft)	[77,78,79]
	AlloDerm	Human cadaveric decellularized dermis; 3D porous matrix of collagen, elastin, and GAGs.	Allows rapid fibroblast infiltration; No donor cells; Low immunogenicity; Revascularization by the host within 2–4 weeks.	Human-derived; No donor site morbidity; Versatility and availability.	Complications and graft shrinkage; High cost.		[80,81]
	Matriderm	Bovine collagen I, III, V + hydrolyzed elastin matrix; Open pore structure; No silicone layer—no need for two-stage procedure.	Promotes fibroblast migration; Increased integration due to structural similarity to native dermis.	Single-stage grafting; Reduced scarring, improved skin elasticity; Rapid vascularization.	High-cost; Thinner than Integra, difficult to handle; Risk of too-fast degradation.		[82,83,84]
	Promogran	55% collagen + 45% oxidized regenerated cellulose (ORC); Freeze-dried matrix; Transforms into a gel in contact with wound fluid.	No living cells; Full biodegradation; Inactivates wound proteases (MMPs).	Effective for DFUs; Promotes a favorable environment for wound healing.	Acellular—no active growth factor delivery; Marginal superiority over standard gauze in randomized clinical trials (RCTs).		[85,86]
Cellular	Apligraf	Bilayer: dermal—bovine collagen type I + neonatal fibroblasts, and epidermal—human keratinocytes.	Metabolically active—own production of matrix proteins; Secretion of growth factors and cytokines; Biodegradable—2–4 weeks.	High healing rate and no donor site necessity; High immunological toleration; FDA-approved for DFUs and venous ulcers.	High-cost; Short shelf life + specific storage requirements; Not for infected wounds.	Matrix is reconstructed from polymers (not preserved native dermis). Allows controlled degradation and cell seeding. Unlike acellular matrices, they are often seeded with living fibroblasts/keratinocytes.	
	OrCel	Bilayer: porous bovine collagen type I sponge seeded with allogeneic fibroblasts—dermal + keratinocytes in pores—epidermal.	Metabolically active—own production of matrix proteins and cytokines; Promotes cell infiltration, neoangiogenesis, and rapid re-epithelialization.	Reduced donor site morbidity; FDA approved for donor sites in patients with epidermolysis bullosa.	High-cost; Limited availability + specific storage requirements; Undeveloped stratum corneum.		[87,88]
	Dermagraft	Human neonatal dermal fibroblasts + polyglactin mesh (biodegradable); Cryopreserved before use.	Fibroblasts are metabolically active and secrete collagen (I, III, VII), growth factors, and cytokines; Reduced immune response.	Rich growth factor environment; Faster healing rates; Off-the-shelf; primarily for chronic diabetic foot ulcers (DFUs).	Cryostorage required; High cost; Multiple applications required for effective results; Specific usage criteria—DFUs.		[89,90,91]
Xenogeneic	EZ-Derm	Aldehyde-crosslinked porcine collagen—decellularized dermis; Dehydrated sheet structured -> room-temperature storage; Non-biodegradable outer surface.	Acellular; Crosslinking reduces antigenicity; Acts as a physical wound cover.	Durable, room-temperature storage; Availability + high shelf life; Effective pain reduction in partial-thickness burns.	Crosslinking impairs bioactivity; Temporary solution, requires removal before grafting.	Porcine collagen is the closest, structurally, to human dermis. Primary role—temporary biological dressings for wound cover, exudate control, and pain reduction. They do not permanently integrate into the host tissue.	[92,93]
	Xe-Derma	Acellular porcine dermis; Maintains the natural collagen + elastin network; Requires rehydration before use.	Minimized risk of adverse immune effects; Biological barrier function; Promotes keratinocyte proliferation.	Facile rehydration and application; Temporary cover, which generally detaches on its own as the wound heals.	High-cost; Reports of adhesion loss or partial decomposition; Limited large RCT data.		[94,95]
	PriMatrix	Acellular fetal bovine dermis; Rich in collagen type I and III; Minimal crosslinking from native ECM; Dehydrated meshed sheets.	Low immunogenic response due to removal of cells, lipids, fats, and carbohydrates; Promotes regenerative healing and remodeling.	Can be used in complex wounds (exposed tendon/bone); Increased bioactivity due to minimal crosslinking; Facile rehydration and application; Long shelf life.	High-cost; Reports of infection and excessive inflammation.		[96,97]
	Oasis Wound Matrix	Matrix—porcine small intestinal submucosa (SIS) + collagen, GAGs, growth factors; Single-layer structure—lyophilized sheet.	SIS retains growth factors and cytokines that stimulate angiogenesis and cell migration; Biodegradable—replaced by host tissue.	Long shelf life—2 years at room temperature; Strong RCT evidence; FDA approved for diabetic/venous ulcers.	Limited to wounds with sufficient vascularization; May require multiple applications.		[93,98,99,100]
Synthetic	Biobrane	Bilayer: silicone membrane + porcine collagen type I-coated nylon mesh; Porous, semi-permeable sheet-like structure.	Collagen coating promotes initial fibrin adhesion; Porosity allows wound exudate drainage; Silicon layer controls a moist environment.	Self-detaches during re-epithelialization; Evidence of efficiency in pediatric burns; Reduced hospital stays.	Not recommended for deep or infected wounds; Specific handling—if not adhered to properly -> risk of infection.	High manufacturing reproducibility, but lacks inherent bioactivity. Growth factor or peptide seeding is required for enhanced cell attachment and biological response.	[101,102,103]
	TransCyte	Bilayer: silicone membrane + porcine collagen type I-coated nylon mesh; Nylon mesh seeded with allogeneic neonatal fibroblasts; Cryopreserved until use.	Accelerate re-epithelialization due to ECM components still in the scaffold; Silicone layer—moisture control.	Growth factor-rich synthetic matrix; Reduces need for autografts; FDA approved for full-thickness and deep partial-thickness burns; Equivalent to human cadaver allograft.	High cost and limited availability; Specific storage requirements.		[104,105,106]
	Suprathel	Copolymer: DL-lactide (>70%), trimethylenecarbonate, ε-caprolactone; Microporous membrane with interconnected pores; Resorbable.	Degrades via hydrolysis—lactic acid release—promotes healing; Acellular; Fast adhesion at body temperature.	Transparency—allows wound monitoring; Significant pain reduction; Often left on until complete healing.	High-cost; Demonstrated efficacy, but slower healing time than grafts; Recommended for superficial or partial-thickness burns.		[107,108,109]
Hybrid	PolyActive	Copolymer: poly(ethylene oxide) (PEO) + poly(butylene terephthalate) (PBT); Porous scaffold seeded with autologous keratinocytes and fibroblasts—interconnected pores.	Synthetic backbone—mechanical stability; Promotes cell attachment.	High mechanical stability + bioactivity; Low immunogenicity risk.	Non-biodegradable component—limits permanent integration; Limited ‘off-the-shelf’ availability due to cellular component.	Scaffolds containing biological ECM components + synthetic polymers, therefore combining the biological signaling of natural materials with the mechanical properties of synthetic materials.	[110,111]
	MySkin	Silicone support with surface coating + autologous keratinocytes.	Surface coating promotes rapid keratinocyte attachment and gives structural stability; The cells are subconfluent.	Reduced culture time due to cell state; Positive reports for DFUs and superficial burns.	Requires donor site; Limited ‘off-the-shelf’ availability due to cellular component.		[110,112]
	TissueTech	Dermal scaffold—hyaluronic-based seeded with autologous fibroblasts (Hyalograft 3D—esterified hyaluronic acid fibers HYAFF) Epidermal replacement—microperforated hyaluronic acid membrane seeded with autologous keratinocytes (Laserskin).	Anti-inflammatory and pro-angiogenic effect from HA; Promotes full-thickness regeneration due to ‘bilayer’ configuration.	Positive reports for the treatment of DFUs in RCLs; Low recurrence rates.	The layers are individual—two product grafting—may lead to complications.		[113,114,115]

**Table 4:** Biological and clinical requirements for dermal substitutes.

	Biological Requirement	Clinical Requirements	Gaps in the Literature	Key Refs.
Burn reconstruction	- 70–200 µm pores for capillary ingrowth; - Vascularization within 14–21 days with a scaffold thickness under 0.4 mm; - Biodegradation vs. tissue maturation; - Reduced contraction.	- Off-the-shelf availability; - Compatible with single- or two- stage protocols; -Antimicrobial tolerance; - Improved scar quality vs. STSG alone.	- No RCTs comparing dermal substitutes; - Limited data on cellular and hybrid substitutes; - Long-term outcomes (>12 months); - Skin appendages restoration unachieved.	[120,121,149,150]
Traumatic soft tissue defects	- Tissue formation over exposed areas (bone, tendon); - Contamination tolerance; - Mechanical resistance over mobile areas; - Limited contracture for functionality purposes; - Controlled biodegradation.	- Single-stage protocols preferred; - Compatible with NPWT; - Rapid functional recovery.	- No RCTs comparing dermal substitutes; - Limited data on cellular and hybrid substitutes; - Lack of standardized outcome; - No comparison between upper and lower extremities.	[126,128,130,151]
Oncologic reconstruction	- Oncological safety; - Integration over avascular sites; - Adjuvant-radiotherapy tolerance; - Adaptability for complex craniofacial defects.	- Two-stage protocol for histological margin confirmation; - Reduced anesthetic time.	- Absent data on cellular and hybrid substitutes; - Radiotherapy interaction not clinically characterized; - No cost analysis for different substitutes; - No systematic evaluation of recurrence.	[135,136,152,153]
Chronic wounds & complex skin defects	- MMP dequestration; - Biofilm tolerance; - Bioactivity; - Repeated application.	- Non-surgical staff application friendly; - Improved wound closure rate as compared to standard-of-care; - Low-cost.	- No RCTs comparing dermal substitutes; - No data available for hybrid substitutes; - Scarce reports on amputation rate, recurrence or patient outcome.	[142,143,148,154]

## Data Availability

No new data were created or analyzed in this study. Data sharing is not applicable to this article.
